# Laser Ablation Study of Cutting Ceramics with Consideration of the Beam Inclination Angle

**DOI:** 10.3390/ma16062509

**Published:** 2023-03-21

**Authors:** Jodok Weixler, Marc Zweifel, Timo Schudeleit, Markus Bambach, Konrad Wegener

**Affiliations:** 1 Institute of Machine Tools and Manufacturing, ETH Zürich, Leonhardstrasse 21, 8092 Zürich, Switzerland; 2 Inspire AG, Technoparkstrasse 1, 8005 Zürich, Switzerland; 3 Advanced Manufacturing Laboratory, ETH Zürich, Technoparkstrasse 1, 8005 Zürich, Switzerland

**Keywords:** laser ablation, tilted irradiation angle, SiAlON, ATZ

## Abstract

Silicon alumina nitride (SiAlON) and alumina toughened zirconia (ATZ) ceramics are applied for ceramic cutting tools to machine, e.g., cast iron, nickel base alloys and other difficult-to-machine materials. The state of the art technology for manufacturing of the cutting tool geometry is grinding. Laser processing of ceramics is already studied in terms of ablation rate and roughness evaluation with the application of dental implant manufacturing. In the present study, laser machining of the mentioned ceramics is explored with a laser beam source of 1064 nm wavelength and 10 ps pulse duration (FWHM). The angle dependent energy specific removal rate is described in a model and the optimal pulse fluence for the different materials and the irradiation angles can be derived. For processing at irradiation angle of up to 75° no decrease of the relative absorption could be observed. For ATZ, lowest surface roughness is determined for both, orthogonal and quasi-tangential processing angle. For SiAlON, the roughness decreases constantly for higher tilt angles. A significant difference in the material answer with change of the sample composition can be detected and the results show the potential of further developing SiAlON ceramics towards machineability for laser ablation.

## 1. Introduction

Ceramic cutting tools bring advantages such as high wear resistance and lower surface roughness on the machined part compared to cemented carbide tools [1]. This leads to a longer lifetime of the tools as well to a higher quality of the machined part. Furthermore, the hardness of ceramic material remains at high level for elevated temperatures [2]. This makes the material interesting for high-speed cutting operations. As ceramics are inert against most work metals [2], they are suitable for machining difficult-to-machine materials such as aluminium or lead-free brass. Due to the brittle-hard nature of ceramics, they are prone to failure due to breakage, as the crack propagation hardly requires any energy. Therefore, a high level of expertise in the use and manufacturing of ceramic tools is necessary to avoid fracture of the blank. Especially the production and use of ceramic micro-tools with a high length to diameter aspect ratio is challenging. This is met on the one hand with the development of fracture-resistant ceramic grades and on the other hand with the development of more gentle and more precise manufacturing processes to reduce mechanical shock and geometric deviations such as run-out. The present study focuses on the fundamentals for developing a new manufacturing process. The current state of the art for processing ceramic cutting edges is grinding [3]. There are two phases of SiAlON ceramics which are relevant for tooling materials. The low temperature β-phase has a high fracture toughness but low thermal shock resistance and relatively low hardness. The high-temperature α-phase has a good wear resistance and hardness but a low fracture toughness [4]. In a recent laser ablation study using a $CO_{2}$ laser at 2 kW with a focal diameter of 0.3 mm it is found that the surface is free from large sized cracks and voids despite some small and few localized micro-cracks [5]. Regarding the fabrication of ceramic cutting tools, pulsed laser radiation at a pulse duration of 12 ps and below and a wavelength of 1064 nm and below is applied in order to add modifications on SiAlON ceramics such as micro textures [6,7]. In these studies flat samples are irradiated orthogonally to the sample surface. Regarding the laser processing of ATZ ceramics, it is reported that the relationship between material removal rate and pulse energy is close to linear. For mixed ceramics it is observed that the threshold fluence is significantly lower compared to single phase alumina or zirconia ceramics [8]. The combination of quasi-tangential processing configuration and 5-axis path planning successfully was applied in a study published by Ackerl at al. [9] for laser machining a component of a dental implant in zirconia. This processing technique enables the shaping of freeform surfaces with low surface roughness compared to the orthogonal laser irradiation. The corresponding principle of calculating the path for synchronous motion of 5 mechanical and two optical axis for the quasi tangential processing configuration is described in detail in an additional publication by Ackerl et al. [10]. However, laser machining in quasi-tangential configuration at high pulse energy leads to cracks on the surface. The quasi-tangential laser machining using a 5-axis kinematic was successfully applied for manufacturing of polycristalline diamond end mills by Warhanek et al. [11] as well as tungsten carbide end mills by Pfaff et al. [12]. A model describing the angle dependent ablation characteristic of fused silica and sapphire was published by Boerner et al. [13]. The introduced ablation model describes the macroscopic ablation volume and ablation efficiency of dielectric materials as a function of the angle of incidence.

The aim of the present study is to describe the inclination angle dependent ablation behaviour of SiAlON and ATZ ceramic in terms of ablation rate and surface quality using a 10 ps pulsed laser. By determining the effective threshold fluence the optimal pulse fluence for lasermachining the ceramic types can be derived as it is given by e-times the threshold fluence [14]. Based on these results the guidelines for designing a quasi-tangential 5-axis laser machining process for manufacturing an end mill geometry are described. This is a fundamental step to establish laser machining as a manufacturing technology for ceramic micro tools with high reliability. The results also give an indication which processing results can be expected on the individual ceramics and if there is some potential for further material development.

## 2. Materials and Methods

In this study, the SiAlON ceramic types LKT 550 and LST 320 as well as the ATZ ceramic type HTZ 500 LC from Ceramtec GmbH are studied. The properties of the machined cylindrical samples are listed in Table 1.

**Figure 1:**
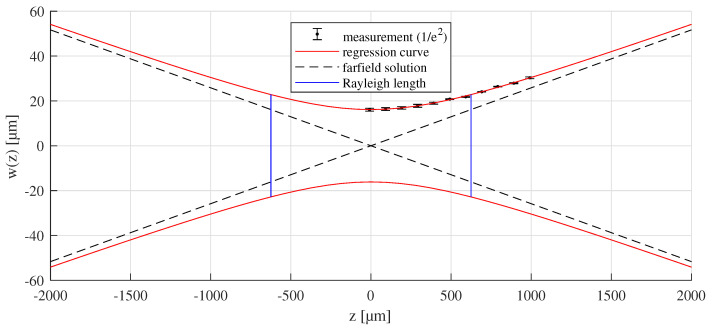
Result of the beam characterisation for a focal length of f = 160 mm revealing a focal radius of $w_{0}\;=\;16.13\;\mu m$, a Rayleigh length of $z_{R}\;=\;624.34\;\mu m$, an opening angle of 1.48° for the farfield solution and an $M^{2}$ of 1.27.

As a laser source, a mode locked solid state laser source emitting at a wavelength of λ=1064nm and a pulse duration of 10 ps (FWHM) is used. The laser beam is deflected by the 2D scan-head intelliscan from SCANLAB GmbH and focused by a telecentric f-theta lens with a focal length of f = 160 mm. In order to determine the longitudinal beam profile in the near field, the Rayleigh length $z_{R}$ and the focal beam radius $w_{0}$, the beam diameter (1/$e^{2}$) is measured at 11 different positions along the beam propagation axis using a commercial 2D beam camera from Ophir. The resulting beam profile is plotted in Figure 1. The peak fluence $\hat{F}$ of a Gaussian laser beam in the focal plane for orthogonal irradiation is calculated according to the following expression [15]:

(1) $$\hat{F}=\frac{2E_{p}}{w_{0}^{2}\cdot \pi }$$

with the pulse energy $E_{p}$. For different irradiation angles $\theta_{i}$, the value of $\hat{F}$ can be calculated according to:

(2) $$\hat{F}\left(\theta_{i}\right)=\frac{2E_{p}\cdot cos\theta_{i}}{w_{0}^{2}\cdot \pi }$$

**Figure 2:**
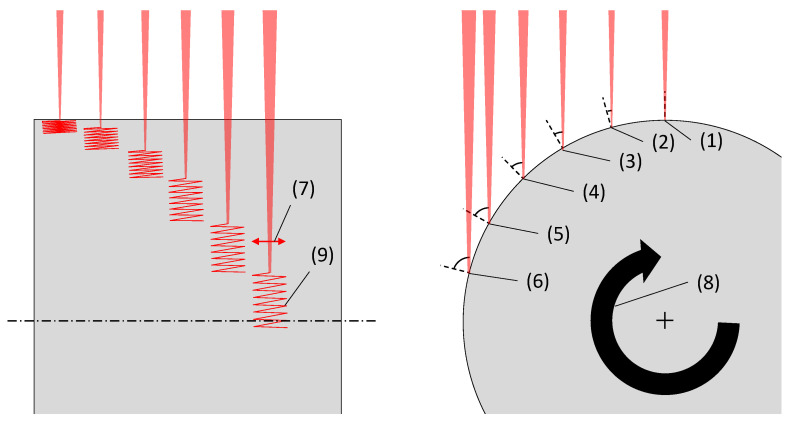
Front view (**left**) and side view (**right**) of the calibration experiment at the different beam incidence configurations from 0° (1) to 75°(6) in 15° intervals. The laserbeam is deflected by a linescan (7) while the cylindrical workpiece is rotated (8) which results in the zig zag scanpath (9).

In order to quantify the angle dependent laser ablation characteristics, a calibration routine on a cylindrical workpiece is performed, similar to the test configuration published by Boerner et al. [13] and Hajri et al. [16]. In the present work a range of irradiation angles from 0° (orthogonal) to 75° in intervals of 15° is tested. The resulting process conditions are illustrated in Figure 2.

The laser process parameters used for the calibration routine are listed in Table 2.

**Figure 3:**
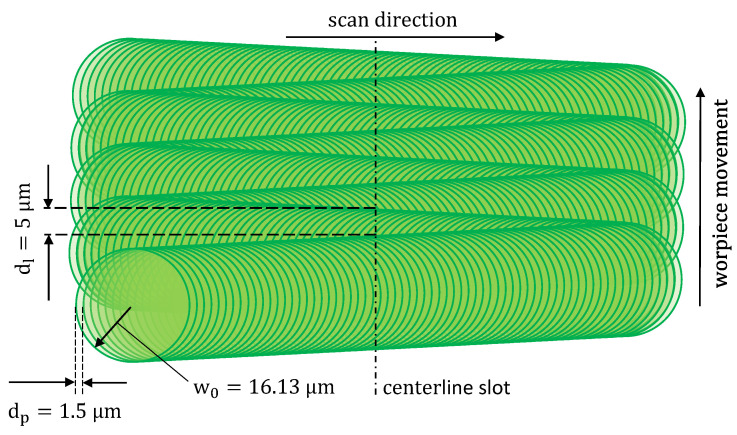
Illustration of the unrolled pulse distribution on the workpiece for the calibration experiment with the parameters listed in Table 1 at orthogonal irradiation.

These parameters lead to the effective pulse to pulse distance of $d_{p}\;=\;1.5\;\mu m$, an average line distance of $d_{l}\;=\;5\;\mu m$ for orthogonal irradiation, present in the center of the slot and a total number of approximately N = $1.51\;\times \;10^{7}$ pulses per machined slot. The unrolled view of the pulse distribution is illustrated in Figure 3. It is mentioned that the bidirectional scan path illustrated in Figure 3 leads to a variation of the line distance along the slot width and the acceleration of the scanner must be considered at the edge of the machined slot. However, these effects can be neglected in the center where the measurement takes place. Here, both the scanspeed and the hatch distance correspond to the desired parameters.

The arithmetic mean height Ra of the machined slots is determined in axial direction of the slot for a measurement length of 0.25 mm by confocal microscopy without using a filter. It is mentioned that the Ra values are only valid for comparison within this study.

The depth of each slot is measured at the center position of each slot at 5 positions around the circumference using confocal microscopy. The ablated volume V for each ablated slot is calculated according to the following equation:

(3) V=(D−t)·l·π·t

with the diameter of the cylinder D, the measured ablation depth t and the width of the machined slot l. Dividing the ablated volume V by the pulse energy $E_{p}$ leads to the energy specific ablation volume for the ablated slot $\eta_{Abl}$:

(4) $$\eta_{Abl}=\frac{V}{E_{p}}$$

These values are determined for all combinations of pulse energy and irradiation angle and are used to describe the angle dependent ablation model [13] by the parameters threshold fluence $F_{th}{(}^{\circ })$, penetration depth $\delta {(}^{\circ })$ and the fitting parameter Θ.

## 3. Results and Discussion

**Figure 4:**
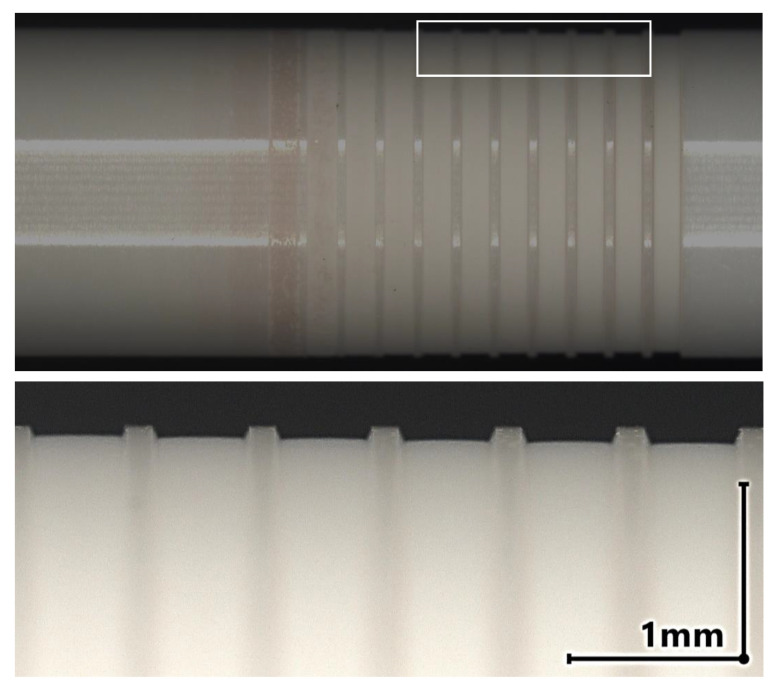
**Top**: Cylindrical ATZ sample HTZ 500 LC with a blank diameter of D = 6 mm after the laser ablation experiment at an angle of irradiation θ = 45°. The pulse energy is increased from $E_{p}\;=\;3\;\mu J$ (left) to $E_{p}\;=\;21\;\mu J$ (right). **Bottom**: Detailed view of the grove profiles machined with a pulse energy from $E_{p}$ = 15 μJ to $E_{p}$ = 20 μJ.

The ablation result on the ATZ ceramic HTZ 500 LC for the irradiation angle $\theta_{i}\;=\;45^{\circ }$ is shown in Figure 4. For $E_{p}\;=\;10\;\mu J$ and higher, volume removal can be detected. This corresponds to $\hat{F}\;=\;1.73\;J\;cm^{-2}$. In the range from $E_{p}\;=\;9\;\mu J$ up to $E_{p}\;=\;12\;\mu J$ ($\hat{F}\;=\;1.56\;J\;cm^{-2}$ to $\hat{F}\;=\;2.08\;J\;cm^{-2}$) a color change to red of the irradiated area is visible which indicates a reduction of oxygen content in the material [10,17]. The bottom of the groove is even at the center, whereas next to the sidewalls a curvature can be observed. This can be attributed to the lower scan speeds caused by the acceleration of the galvo scanner.

**Figure 5:**
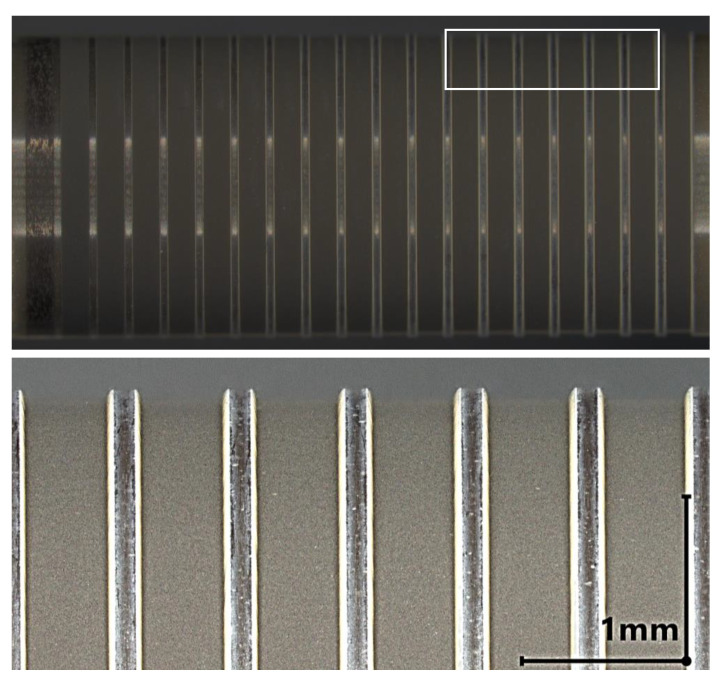
**Top**: Cylindrical SiAlON sample LKT 550 with a blank diameter of D = 6 mm after the laser ablation experiment at an angle of irradiation θ = 75°. At $E_{p}\;=\;3\;\mu J$ (left) hardly any material removal can be detected but a change in surface is visible. **Bottom**: Detailed view of the groves machined with a pulse energy from $E_{p}$ = 15 μJ to $E_{p}$ = 20 μJ.

**Figure 6:**
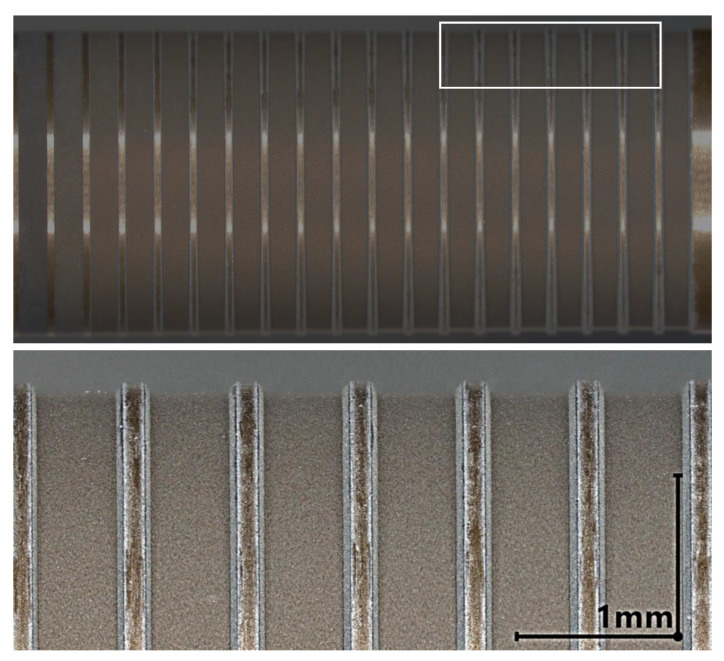
**Top**: Cylindrical SiAlON sample LST 320 with a blank diameter of D = 6 mm after the laser ablation experiment at θ = 75°. Material removal can be detected for the whole range of $E_{p}$. **Bottom**: Detailed view of the groves machined with a pulse energy from $E_{p}$ = 15 μJ to $E_{p}$ = 20 μJ.

The ablation result of the SiAlON ceramic samples LKT 550 and LST 320 at a angle of irradiation $\theta_{i}$ = 75° is shown in Figure 5 and Figure 6, respectively. In both cases the surface of the ablated region on the bottom of the groove appears grainy. At the edge region of the groove some white debris is visible for both SiAlON types. This can be attributed to Si3N4-decomposition and Si-oxidation [18] as the laser ablation experiment was performed in ambient atmosphere. For the latter the chemical reaction can be described according to:

(5) $$Si_{3}N_{4}+3\;O_{2}\to 3\;SiO_{2}+N_{4}$$

**Figure 7:**
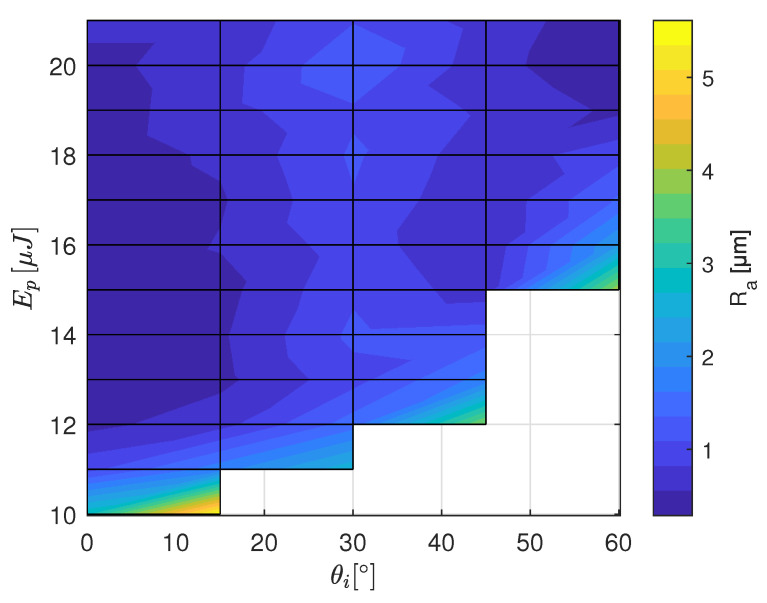
Ra values measured on the ATZ ceramic HTZ 500 LC for the range of pulse energies and the tilt angles from the calibration experiment (see Figure 2). The white area indicates where no ablation was observed.

**Figure 8:**
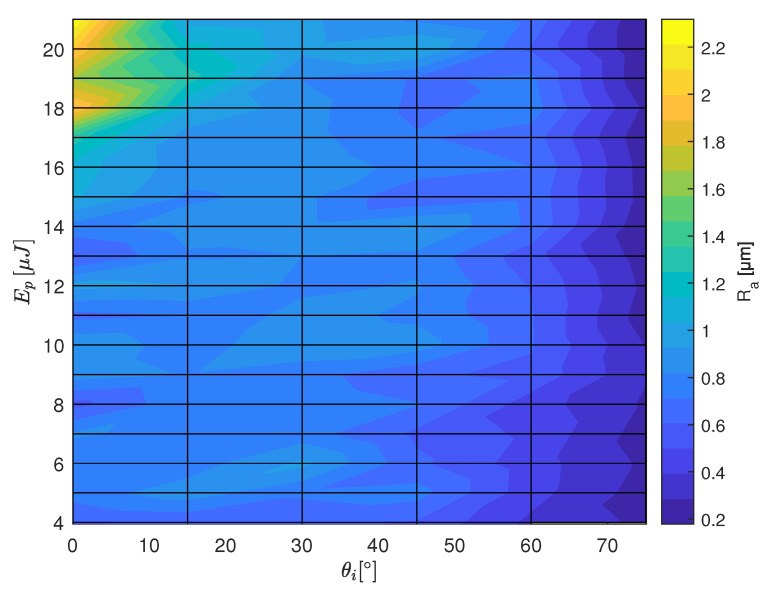
Ra values measured on the SiAlON ceramic LKT 550 for the range of pulse energies and the tilt angles from the calibration experiment (see Figure 2).

**Figure 9:**
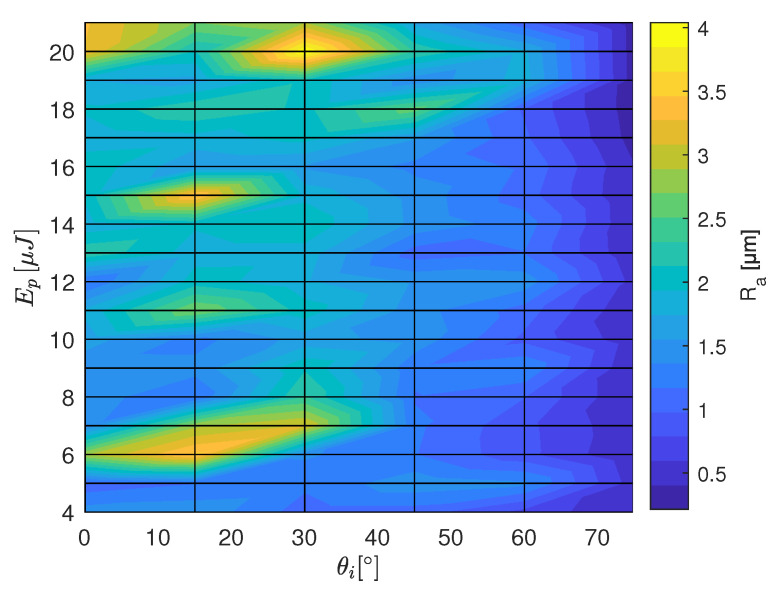
Ra values measured on the SiAlON ceramic LST 320 for the range of pulse energies and the tilt angles from the calibration experiment (see Figure 2).

The results of the roughness measurement are plotted in Figure 7, Figure 8 and Figure 9 as a function of the laser irradiation angle and the pulse energy. In the case of of HTZ 500 LC high roughness values are measured for energy density close to the ablation threshold. It implies that the ablation process in this energy regime is unsteady. This could be explained by the highly nonlinear laser absorption mechanism including avalanche ionisation, multi-photon absorption and tunneling ionisation [19] together with the defect dependent absorption of laser energy in dielectrics [20]. This can lead to selective removal which as a final result leads to an undulating surface profile. Around $\theta_{i}\;=\;30^{\circ }$ high Ra values are determined for the full range of $E_{p}$. Interestingly, the lowest Ra values are measured for both, the orthogonal irradiation and close to tangential irradiation if $\hat{F}$ is at least 1.2 times the lower limit for ablation.

In the case of LKT 550 high $E_{p}$ and orthogonal irradiation angle lead to high Ra values. This can be explained by the high $\hat{F}$ leading to re-condensation, Si3N4-decomposition and Si-oxidation [18]. Low Ra values are measured for the quasi-tangential laser irradiation. The Ra values on the LST 320 samples show a similar characteristic regarding the $\theta_{i}$ dependence. Comparing the absolute values, the Ra values are about two times higher compared to LKT 550. As the roughness values are in the order of the grain size it is very likely that thermo-mechanical shock of the laser pulse causes pullout of full grains.

**Figure 10:**
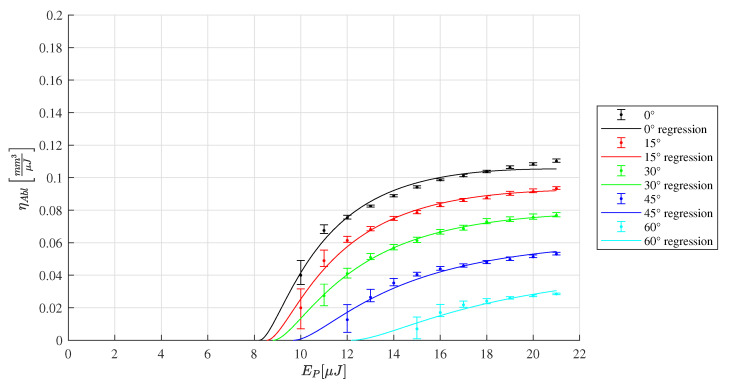
Ablation efficiency according to the depth measurement of the ablated of HTZ 500 LC samples for a range of inclination angles from $0^{\circ }$ to $60^{\circ }.$ The corresponding ablation model according to [13] leads to the parameters $F_{th}\left(0^{\circ }\right)\;=\;1.29\;J\;cm^{-2}$ and $\delta (0^{\circ })\;=\;357\;nm$.

**Figure 11:**
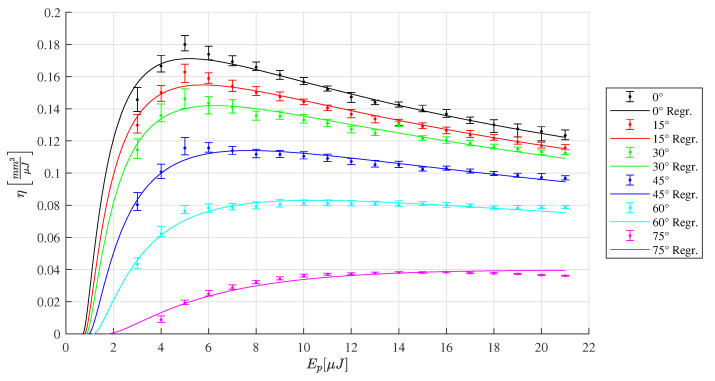
Ablation efficiency of LKT 550 for different inclination angles ranging from $0^{\circ }$ to $75^{\circ }.$ The corresponding regression of the ablation model [13] leads to the parameters $F_{th}\left(0^{\circ }\right)=1.18\;J\;cm^{-2}$ and $\delta (0^{\circ })\;=\;70\;nm$.

**Figure 12:**
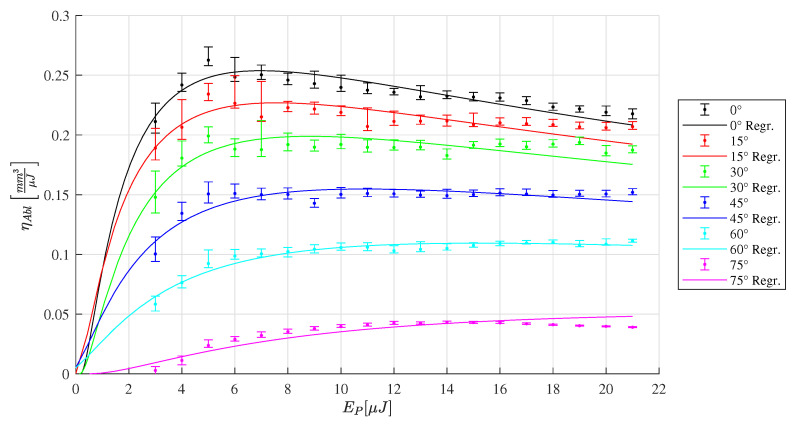
Energy specific ablation volume LST 320 for a range of inclination angles ranging from $0^{\circ }$ to $75^{\circ }.$ The corresponding regression of the ablation model [13] leads to the parameters $F_{th}\left(0^{\circ }\right)=0.26\;J\;cm^{-2}$ and $\delta (0^{\circ })\;=\;139\;nm$.

The depth of the machined slots is analyzed by confocal microscopy. The energy specific ablation volume $\eta_{Abl}$ is calculated by Equation (4) and plotted in Figure 10, Figure 11 and Figure 12. For each radiation angle, the ablation efficiency can be determined for the range of pulse energies listed in Table 2. Based on this data, the threshold fluence and penetration depth are determined [13] for HTZ 500 LC

(6) $$F_{th}\left(0^{\circ }\right)=1.29\;J\;cm^{-2}$$

(7) $$\delta (0^{\circ })\;=\;357\;nm$$

for LKT 550

(8) $$F_{th}\left(0^{\circ }\right)=1.18\;J\;cm^{-2}$$

(9) $$\delta (0^{\circ })\;=\;70\;nm$$

and for LST 320

(10) $$F_{th}\left(0^{\circ }\right)=0.26\;J\;cm^{-2}$$

(11) $$\delta (0^{\circ })\;=\;139\;nm.$$

For HTZ 500 LC the resulting $F_{th}\left(0^{\circ }\right)$ and $\delta (0^{\circ })$ seem reasonable as they are comparable to the values determined in a different study on zirconia-based ceramics [21]. It is mentioned that the $F_{th}\left(0^{\circ }\right)$ value is lower compared to the peak fluence $\hat{F}\left(0^{\circ },10\;\mu J\right)\;=\;2.2\;J\;cm^{-2}$ for which volume removal is detected in this experiment. This could be explained by the peak fluence not beeing sufficient for material removal. According to the results measured within the range of this study, it is beneficial to increase the energy density to maximize the $\eta_{Abl}$. This observation is consistent with the statement that intensity is significantly responsible for laser absorption in zirconia-based ceramics [22]. However, it is suspected that the maximum $\eta_{Abl}$ is not achieved within the tested range of $E_{p}$ in this study and higher pulse energies could further increase the $\eta_{Abl}$ value.

In the case of the SiAlON ceramic LKT 550 the highest energy specific ablation volume of $\eta_{Abl}\;=\;0.18\;mm^{3}\;\mu J^{-1}$ is determined at the orthogonal laser irradiation for a pulse energy of 5 μJ (see Figure 11). In contrast to the measurement of HTZ 500 LC, the plot of LKT 550 and LST 320 reveal a pronounced maximum for the $\eta_{Abl}$ value and drop for pulse energy values above 5μJ. It can be derived for the SiAlON ceramics that it is useful to not exceed the fluence level for which the maximum energy specific ablation volume is determined to reduce both, the surplus energy input and the machining time. For the LST 320 slots, the highest $\eta_{Abl}$ can be determined compared to the other ceramic types tested in this study.

**Figure 13:**
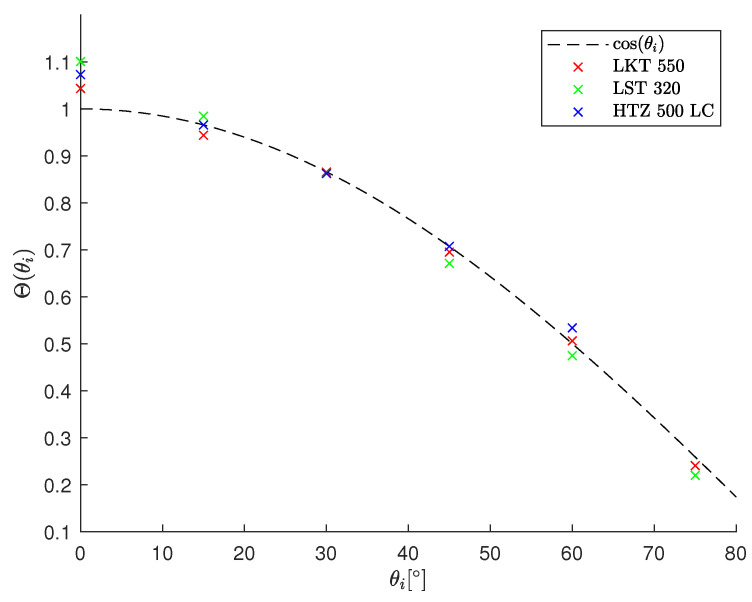
Plot of the fitting parameter Θ for the ablation experiment of HTZ 500 LC, LKT 550 and LST 320. The plot reveals that the relative absorptance is nearly equal to 1. Deviations can be explained by plasma and particle shielding at a low angle of incidence. The relative absorptance A is normalized by the absolute absorptance of all tested ceramics at 30°.

The resulting values of Θ for the individual tilt angles θ are plotted in Figure 13 for the three ceramics. The values are close to the cosine function which means the relative absorption is close to 1 up to an irradiation angle of 75°. Deviations at the orthogonal processing configuration can be explained by particle shielding as the time between two pulses is 2.5 μs and the directional ejection of particles lasts up to several 10 μs [21]. There is a trend that LST 320 deviates the most from the cosine function. Especially at low irradiation angle this is an indicator that the particel shielding is the most pronounced for this material.

## 4. Application

**Figure 14:**
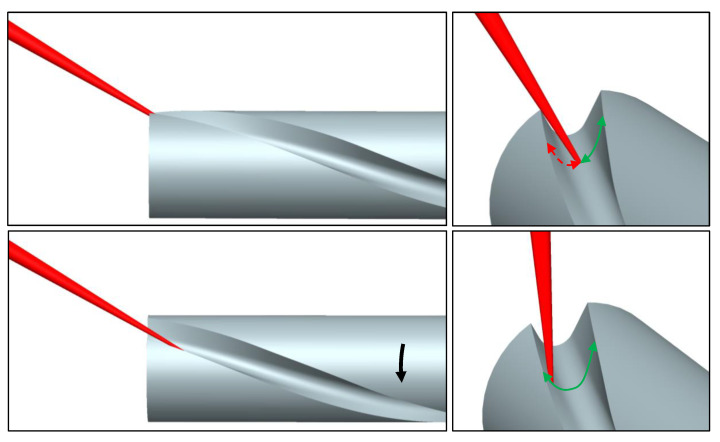
Visualisation of the beam-workpiece intersection resulting from the helix angle of the groove (**top**). Because of self-shading, a part of the cross section cannot be accessed (red arrow). **Bottom**: By rotation of the scan path around the cylindrical axis (black arrow) the full range of the groove can be accessed by the scan path (green).

In the following, the procedure for designing a quasi-tangential 5-axis laser machining process for manufacturing an end mill geometry is described. Depending on the helix angle of the tool, the scanprofile needs to be rotated arround the c-axis, to avoid intersection of the beam with the not ablated volume part as displayed in Figure 14.

Furthermore, it needs to be ensured, that the laser contact angle $\theta_{i}$ does not exceed the limit for ablation. This would lead to a taper angle reducing the cross sectional area along the axial direction. From the experimental results in this study the angle dependent relative absorption can be assumed to be constant for the tested ceramics. From this, the ablation limit can be written as a function of two tilt angles β and gamma γ, representing the swivel angle of the B-axis and the tilt due to the slope of the groove, respectively

(12) $$\hat{F}=\frac{2E_{p}\cdot cos\;\beta \cdot cos\;\gamma }{w_{0}^{2}\cdot \pi }$$

The limiting angle due to the slope of the groove γ tilt angle of the ablation limit can be written as a function of the swivel angle β.

(13) $$\gamma =arccos\left(\frac{\hat{F}\cdot w_{0}^{2}\cdot \pi }{2E_{p}\cdot cos\;\beta }\right)$$

This defines the lower limit for the opening angle of the profile which is going to be ablated. More precisely, the angle between the tangent of the end of the scan-profile must not fall below two times γ, because the increase of the spot area would cause a reduction of the fluence below the ablation limit and lead to a taper angle along the groove profile.

## 5. Conclusions

The ATZ ceramic HTZ 500 LC and the SiAlON ceramics LKT 550 and LST 320 are studied regarding the removal rate and the surface roughness after pulsed laser machining at different irradiation angles for the first time using a pulse duration of 10 ps and a wavelength of 1064 nm. For both SiAlON ceramics, decomposition and oxidation are present from $\hat{F}\;=\;2.45\;J\;cm^{-2}$ in the case of LKT 550 and from $\hat{F}\;=\;2.20\;J\;cm^{-2}$ in the case of LST 320. Below this fluence level, surface roughness can be minimized by a higher irradiation angle. Changing the irradiation angle from $\theta_{i}\;=\;45^{\circ }$ to $\theta_{i}\;=\;75^{\circ }$ leads to a reduction of Ra by a factor of 3 for the whole range of $E_{p}$ for both SiAlON ceramics. For the ATZ ceramic, coloration as an indicator for reduction of oxigen content in the material is observed in the range from $\hat{F}\;=\;1.56\;J\;cm^{-2}$ to $\hat{F}\;=\;2.08\;J\;cm^{-2}$. At $\theta_{i}\;=\;30^{\circ }$ relatively high Ra values are determined for all tested $E_{p}$. Interestingly, the lowest Ra values are measured for both, the orthogonal irradiation and close to tangential irradiation if $\hat{F}$ is about 1.2 times higher than the lower limit for ablation. Regarding the SiAlON ceramics it is recommended to split the machining process into a roughening operation at orthogonal irradiation and a finishing operation at high tilt angle to achieve both, productivity and quality the development. In the case of HTZ 500 LC it can be stated that both, highest productivity and low surface roughness can be achieved at orthogonal irradiation and up to $E_{p}$ = 20 μJ. In this case splitting of the machining operation would not be required. The ablation depth of the tested ceramics can be described for different $\theta_{i}$ by a model [13] with the parameters $F_{th}\left(0^{\circ }\right)$, $\delta (0^{\circ })$, Θ and ΔE. From these values also the optimal fluence for setting up a laser process can be derived as it is given by e-times the threshold fluence [14]. From the ablation volume it can be derived that increasing $\theta_{i}$ up to $\theta_{i}\;=\;75^{\circ }$ does not significantly increase the relative reflection. Following this result, the limiting slope for the scan profile of the groove can be expressed as a function of the swivel angle of the cylinder. Regarding the suitability of the three materials for laser machining operation, low removal rates have to be expected for the ATZ ceramic and the irradiation angle of 30${}^{\circ }$ should be avoided in order to reduce the surface roughness. For the SiAlON ceramic, high roughness values in the order of the grain size of the material are present for the orthogonal machining configuration. This indicates that the removal mechanism involves pull-out of complete grains. To enhance the laser machining quality of SiAlON ceramics at orthogonal irradiation angle, this effect needs to be avoided. This shows the potential in developing SiAlON ceramics with a higher thermo-mechanical shock resistance for example by increasing the portion of the α-phase in the material.

## Figures and Tables

**Table 1:** Physical properties of the treated ceramics.

Indication	Symbol	LKT 550	LST 320	HTZ 500 LC
description		SiAlON	SiAlON (20 % TiN)	ATZ ceramic
density	ρ	3.24 $g\;cm^{-3}$	3.53 $g\;cm^{-3}$	5.96 $g\;cm^{-3}$
Vickers hardness	HV 10	17.5 GPa	16.6 GPa	13 GPa (HV1)
4-point bending strength	$\sigma_{4B}$	1020 MPa	850 MPa	1858 MPa
fracture toughness	$K_{IC}$	$6.5\;MPa\;\sqrt{m}$	$6.2\;MPa\;\sqrt{m}$	$11\;MPa\;\sqrt{m}$

**Table 2:** Process parameters for the laser calibration method.

Parameter		Unit	Value
wavelength	λ	[nm]	1064
pulse duration	$\tau_{p}$	[ps]	10
focal radius	$w_{0}$	[μm]	14.4
tilt angle	θ	[°]	0–75
pulse energy	$E_{p}$	[μJ]	3–21
repetition rate	$f_{rep}$	[kHz]	400
scanspeed	$v_{s}$	[m/s]	0.6
width of slot	*l*	[mm]	0.6
rotational speed	ω	[rad/min]	100
number of turns	R	[-]	10
number of pulses	N	[-]	$1.51\times 10^{7}$

## Data Availability

The datasets generated during and/or analyzed during the current study are available from the corresponding author on reasonable request.
